# Estimation of the incidence of MRSA patients: evaluation of a surveillance system using health insurance claim data

**DOI:** 10.1017/S0950268816000674

**Published:** 2016-04-08

**Authors:** S. TANIHARA, S. SUZUKI

**Affiliations:** 1 Department of Public Health and Preventive Medicine, School of Medicine, Fukuoka University, Fukuoka, Japan; 2 Department of Bacteriology II, National Institute of Infectious Diseases, Tokyo, Japan

**Keywords:** Disease notification, health insurance claims, MRSA, sentinel surveillance

## Abstract

Because sentinel surveillance systems cannot obtain information about patients who visit non-sentinel medical facilities, the characteristics of patients identified by these systems may be biased. In this study, we evaluated the representativeness of a methicillin-resistant *Staphylococcus aureus* (MRSA) surveillance system using health insurance claim (HIC) data, which does not depend on physician notification. We calculated the age-specific incidence of MRSA patients using data from the Japan Nosocomial Infections Surveillance (JANIS) programme, which is based on sentinel surveillance systems, and inpatient HICs submitted to employee health insurance organizations in 2011, and then computed age-specific incidence ratios between the HIC and JANIS data. Age-specific MRSA incidence in both datasets followed J-shaped curves with similar shapes. For all age groups, the ratios between HIC and JANIS data were around 10. These findings indicate that JANIS notification of MRSA cases was not affected by patients’ age.

## INTRODUCTION

Methicillin-resistant *Staphylococcus aureus* (MRSA) has become prevalent worldwide, limiting therapeutic options and posing a significant threat to medical care [1]. Surveillance is a critical first step in obtaining basic statistics on MRSA, including the number of affected patients, as well as in implementation of effective treatment protocols and infection control measures [2]. MRSA is a common drug-resistant bacterium, and the sentinel systems used for MRSA surveillance require specific medical facilities to provide alerts when patients meet specified diagnostic criteria. Most sentinel medical facilities are university hospitals [3] or large, tertiary educational hospitals [4]. Only a few surveillance systems [5] mandate the participation of all medical facilities or community hospitals [6] in a specific area.

Since sentinel surveillance systems cannot obtain information on patients who visit non-sentinel medical facilities, these systems underestimate the actual number of patients and the characteristics of identified patients may be biased. For instance, it was reported that the sentinel measles surveillance system underestimated the number of adult measles cases in 2007 in Japan [7]. The reliability of surveillance systems should be monitored periodically using data that are separate from the surveillance sources. However, the evaluation of MRSA surveillance systems using such data has been limited [8–10].

The Japan Nosocomial Infections Surveillance (JANIS) system is a national surveillance programme organized by the Ministry of Health, Labour and Welfare (MHLW) of Japan, and is designed to provide basic information on the incidence and prevalence of nosocomial infections and antimicrobial-resistant bacteria in Japanese medical settings. JANIS was launched in 2000 and now comprises the following five divisions: clinical laboratory (CL), antimicrobial-resistant bacterial infection (ARBI), intensive-care unit (ICU), surgical site infection (SSI), and neonatal intensive-care unit (NICU). Because participation in the JANIS sentinel surveillance system is on a voluntary basis, the characteristics of its sentinel medical facilities may be biased. For example, medical facilities with <200 beds were not eligible to participate in JANIS until 2013. In 2011, there were 2052 eligible hospitals with ⩾200 beds, and 495 (23·8%) of these participated in JANIS. Although JANIS is a sentinel surveillance system, for which the characteristics of reported patients may differ from those of cases overall, no previous studies have evaluated the representativeness of the data submitted to JANIS.

In Japan, insurers possess comprehensive information regarding their subscribers' medical procedures, because health insurance coverage is universal and was originally based on fee-for-service reimbursement. There are three major public health insurance systems in Japan: the medical system for elderly citizens aged ⩾75 years, National Health Insurance, and employees' insurance (employees working in a small company and their dependants are covered by the Japan Health Insurance Association, whereas large companies are able to operate health insurance organizations to provide health insurance for their employees).

In order to claim reimbursement for the costs of healthcare services provided in a given calendar month, each healthcare provider submits health insurance claims (HICs) to the Health Insurance Claims Review & Reimbursement Services (HICRRS) or to the National Health Insurance Organization (NHIO), depending on the patient's specific health insurance plan. The HICRRS or NHIO then investigates the HICs to determine each patient's eligibility for coverage and whether the services provided conform to the reimbursement rules defined by the MHLW. After these investigations, the HICRRS or NHIO then sends the HICs to the insurers. The format of HICs is uniform, and the information they contain is not affected by healthcare providers' notifications to surveillance systems such as JANIS. Therefore, HICs have been used for a variety of purposes including evaluation of adult measles surveillance in Japan [7], measurement of the length of stay of hospitalized patients and their associated antibiotic costs [11], and assessment of the association between hospital case volume and mortality in non-elderly pneumonia patients [12]. However, HICs have not previously been utilized to assess the surveillance systems for drug-resistant bacteria such as MRSA. In this study, we used HIC data to evaluate the MRSA surveillance system of JANIS and to estimate the incidence of MRSA patients in Japan.

## METHODS

### HICs and definition of MRSA patients

This study investigated all inpatient HICs submitted from hospitals certified for diagnosis-procedure combination per-diem payment system (DPC/PDPS), as well as HICs from hospitals not certified for DPC/PDPS, to employee health insurance organizations from April 2011 to March 2012. The number of insured persons and their dependants was 1 475 524 as of 31 March 2012. In the HIC data, a MRSA patient was defined as a person who received at least one injected antibiotic for MRSA, including vancomycin (VCM), teicoplanin (TEIC), arbekacin (ABK), linezolid (LZD), and daptomycin (DAP).

Due to reimbursement regulations in Japan, healthcare providers are required to submit a single HIC combining all healthcare services rendered by the provider for each patient in a given calendar month; therefore, for example, two HICs are issued if a patient is hospitalized from 20 October to 8 November. If a patient was hospitalized for ⩾2 months without interruption, all of their inpatient HICs were collected; we identified all HICs issued on behalf of the patient and investigated injection of antibiotics for MRSA during the hospitalization.

### JANIS programme

The methodology of JANIS has been described in detail elsewhere [13, 14]. In this study, we used the number of MRSA patients submitted to the ARBI division. Based on CLSI2007 (M100-S17) [15], JANIS defines MRSA as bacterial resistance to oxacillin (MPIPC) or cefoxitin (CFX). A MRSA patient was defined as an inpatient from whom MRSA was isolated, to whom anti-MRSA antibiotics were prescribed, and in whom inflammation was observed either at the infection site or via a positive biomarker assay.

### Estimation of MRSA incidence and statistical analysis

We calculated the age-specific incidence (per 100 000 person-years) of MRSA inpatients using data separately from both HICs and JANIS. The numerator was the number of MRSA patients identified from HICs or submitted to the ARBI division of JANIS. For the HIC data, the denominator was the number of persons insured by the health insurance organizations. For JANIS, assuming a census population as source population, it was the population on 1 April 2012, as estimated by the Ministry of Internal Affairs and Communications based on the 2010 Population Census [16] because JANIS is a national surveillance programme organized by the MHLW of Japan. Next, we compared the age-specific incidence ratio between the two groups and calculated the age-adjusted incidence ratio using the Mantel–Haenszel method. A two-tailed *P* value <0·05 was considered statistically significant. All analyses were performed using IBM SPSS Statistics v. 19 (IBM Corp., USA).

### Ethical concerns

Personally identifiable information in HICs was removed prior to the analysis using the MediC4 encoding system (Japan Medical Data Center, Tokyo, Japan) [17]. We used only the aggregated results of JANIS data tables and could not identify any specific individuals. The study protocol was approved by the Ethics Committee of Fukuoka University.

## RESULTS

Table 1 shows the number of persons insured by health insurance organizations (hereafter, insured persons) and the estimated population from the 2010 Population Census by the Ministry of Internal Affairs and Communications in Japan, and the age-stratified proportion of insured persons. The number of insured persons peaked in the 30–39 years age group, which accounted for 21·4% of the total. About half (56·3%) of insured persons were aged 20–49 years. The proportion of insured persons relative to the total population of Japan was 1·16%, and tended to decline with age. The highest proportion of insured persons (2·09%) was in the 0–4 years age group. For all groups aged <59 years, the proportion of the number of insured persons relative to the total population of Japan was >1·0%. However, the proportions in age groups 60–69 and ⩾70 years were 0·32% and 0·03%, respectively.

**Table 1.** The number of persons insured by health insurance organizations and the estimated population in Japan

Age group (years)	Number insured* (A)	(%)	Population in Japan† (B)	(%)	A/B (%)
0–4	110 425	(7·5)	5 289 000	(4·1)	2·09
5–9	105 797	(7·2)	5 452 000	(4·3)	1·94
10–19	198 983	(13·5)	11 959 000	(9·4)	1·66
20–29	239 403	(16·2)	13 454 000	(10·5)	1·78
30–39	315 081	(21·4)	17 509 000	(13·7)	1·80
40–49	276 187	(18·7)	17 420 000	(13·7)	1·59
50–59	162 707	(11·0)	15 744 000	(12·3)	1·03
60–69	59 769	(4·1)	18 432 000	(14·4)	0·32
⩾70	7172	(0·5)	22 308 000	(17·5)	0·03
Total	1 475 524	(100·0)	127 567 000	(100·0)	1·16

* The number of persons insured by health insurance organizations is based on data at 31 March 2012.

† The population on 1 April 2012, was estimated by the Ministry of Internal Affairs and Communications based on the 2010 Population Census.

Table 2 shows the age-grouped numbers of MRSA patients from the HIC database and those reported to the JANIS surveillance system. We identified 537 (male: 329, 61·3%; female: 208, 38·7%) inpatients who received at least one injected antibiotic for MRSA, including VCM, TEIC, ABK, LZD, and DAP. The age groups showed a bimodal distribution, with the 50–59 years age group demonstrating the highest number of MRSA patients (111, 20·7%). This was followed sequentially by age groups 0–4 years (102, 19·0%), 60–69 years (90, 16·8%), and 40–49 years (88, 16·4%). The smallest group was aged 5–9 years (6, 1·1%), followed by 10–19 years (27, 5·0%), ⩾70 years (28, 5·2%), and 20–29 years (36, 6·7%).

**Table 2.** The number of MRSA patients defined by HICs or reported to JANIS

Age group (years)	HICs	(%)	JANIS	(%)
0–4	102	(19·0)	528	(3·2)
5–9	6	(1·1)	53	(0·3)
10–19	27	(5·0)	133	(0·8)
20–29	36	(6·7)	190	(1·1)
30–39	49	(9·1)	284	(1·7)
40–49	88	(16·4)	567	(3·4)
50–59	111	(20·7)	1009	(6·1)
60–69	90	(16·8)	2746	(16·6)
⩾70	28	(5·2)	11 067	(66·8)
Total	537	(100·0)	16 577	(100·0)

HIC, Health insurance claims; JANIS, Japan Nosocomial Infections Surveillance system; MRSA, methicillin-resistant *Staphylococcus aureus.*

The total number of MRSA patients reported to JANIS was 16 577 (male: 10811, 65·2%; female: 5766, 34·8%). In both datasets, the proportion of male patients was about 60%. These patients' ages also showed a bimodal distribution; however, the distribution differed from that of the HIC data. The ⩾70 years age group was the largest (11067, 66·8%), containing about two-thirds of the total MRSA patients. This was followed by age groups 60–69 years (2746, 16·6%), 50–59 years (1009, 6·1%), and 40–49 years (567, 3·4%). As with the HIC data, the smallest group was aged 5–9 years (53, 0·3%). The number of reported MRSA patients increased with age, except in the 0–4 years age group.

Table 3 shows the age-specific incidence of MRSA patients in 2012 for both the HIC and JANIS data, as well as the age-specific incidence ratios between the HIC and JANIS data. Both groups showed a bimodal distribution. For persons insured by the health insurance organizations, those aged ⩾70 years had the highest incidence (390·4/100 000 person-years). This was followed by age groups 60–69 years (150·6/100 000), 0–4 years (92·4/100 000), and 50–59 years (68·2/100 000). The lowest incidence was in the 5–9 years age group (5·7/100 000). The age-specific distribution of MRSA incidence for the JANIS data was similar to that based on HICs. The highest incidence was observed in the ⩾70 years age group (49·6/100 000 person-years), followed by age groups 60–69 years (14·9/100 000), 0–4 years (10·0/100 000), and 50–59 years (6·4/100 000). The lowest incidence was in the 5–9 years age group (1·0/100 000). The crude incidence ratio was 2·8 [95% confidence interval (CI) 2·6–3·1]. However, except for the 5–9 years age group, the age-specific incidence ratios between the HIC and JANIS data were around 10. The age-adjusted incidence ratio, determined by the Mantel–Haenszel method, was 9·9 (95% CI 9·0–10·8).

**Table 3.** Age-specific MRSA incidence (per 100 000 person-years) for HIC and JANIS data and incidence ratios between HIC and JANIS data

Age group (years)	Incidence (95% CI)				Incidence ratio (95% CI)	
	HICs		JANIS			
0–4	92·4	(76·1–112·1)	10·0	(9·2–10·9)	9·2	(7·5–11·4)
5–9	5·7	(2·6–12·4)	1·0	(0·7–1·3)	5·7	(2·5–13·6)
10–19	13·6	(9·3–19·7)	1·1	(0·9–1·3)	12·4	(8·1–18·5)
20–29	15·0	(10·9–20·8)	1·4	(1·2–1·6)	10·7	(7·5–15·2)
30–39	15·6	(11·8–20·6)	1·6	(1·4–1·8)	9·8	(7·1–13·0)
40–49	31·9	(25·9–39·3)	3·3	(3·0–3·5)	9·7	(7·8–12·3)
50–59	68·2	(56·7–82·1)	6·4	(6·0–6·8)	10·7	(8·8–12·9)
60–69	150·6	(122·5–185·1)	14·9	(14·4–15·5)	10·1	(8·2–12·5)
⩾70	390·4	(270·1–564·3)	49·6	(48·7–50·5)	7·9	(5·4–11·4)
Total	36·4	(33·4–39·6)	13·0	(12·8–13·2)	2·8	(2·6–3·1)
Age adjusted					9·9	(9·0–10·8)

CI, Confidence interval; HICs, health insurance claims; JANIS, Japan Nosocomial Infections Surveillance system; MRSA, methicillin-resistant *Staphylococcus aureus.*

Age-adjusted incidence ratio was estimated by the Mantel–Haenszel method.

## DISCUSSION

In this study, we calculated and compared the incidences of MRSA patients based on HIC data for those insured by health insurance organizations, as well as data reported to JANIS. There are two major findings. The first is that the age-specific incidence of MRSA patients showed a J-shaped curve for both the HIC and JANIS data. The second is that the age-specific incidence ratios for the HIC and JANIS data were around 10 for almost all age groups. Our results suggest that direct notification of JANIS regarding MRSA patients is not affected by patients' age, and that information from HICs is useful for evaluation of the sentinel infection surveillance system.

For both HICs and JANIS, the age-specific incidence of MRSA patients showed a J-shaped curve and the incidence was lowest in the 5–9 years age group. MRSA colonization has been observed in healthy children [18] and the elderly living in retirement homes [19] or nursing homes [20, 21]. Risk factors for mortality in patients with *Staphylococcus aureus* bacteraemia include malignancy, low serum albumin, high glucose, and methicillin resistance [22]. MRSA pneumonia is associated with significant mortality [23], and patients treated with injected anti-MRSA drugs generally have severe diseases that precede MRSA infection. Finally, a J-shaped curve characterizes the age-specific mortality in malignant neoplasms, pneumonia, and circulatory diseases in Japan [24]. It is therefore not surprising to find a J-shaped curve in the age-specific incidence of MRSA patients.

The MRSA patients' age-specific incidence ratios calculated from both the HIC and JANIS data were almost constant. Because of the universal health insurance coverage system in Japan, all information about healthcare services received by MRSA inpatients is provided to insurers. During the study period, medical facilities with <200 beds were not eligible to participate in JANIS. In 2011, of 2052 eligible hospitals with ⩾200 beds, 495 (23·8%) participated in JANIS. Therefore, the number of MRSA patients reported to JANIS underestimates the true number of MRSA patients in Japan. One of the reasons for the difference in age-specific incidences derived using HIC and JANIS data is that JANIS is a voluntary surveillance system, and not all medical facilities are registered as sentinels.

There are three major advantages of using HICs for infection surveillance. The first is that the information HICs contain is not affected by healthcare providers' notifications to surveillance systems [7]. The second is that the data are readily available and collected at low cost because the format of HICs in Japan's health insurance system is uniform and computerized [11,12]. The third is that the use of HICs eliminates inadvertent data duplications because insurers can identify whether patients are treated at multiple medical facilities for the same disease [7]. However, timeliness has not been taken into consideration. Since healthcare providers are required to submit, for each patient treated, a single HIC combining all healthcare services offered by the provider in a given month, the data from an HIC is not necessarily timely for surveillance systems.

HIC data defines MRSA patients differently from JANIS. All inpatients who receive at least one injected antibiotic for MRSA are identified as MRSA patients by HICs, and drug resistance is not considered. Recently, a guideline for the restricted use of anti-MRSA medications [25–28] was applied, and assessment of drug resistance is recommended before such medications are used [25–28]. However, the definition of MRSA based on injected antibiotics is not specific, and it is possible that any given infection might be caused by a pathogen other than MRSA. For example, a physician who is not an infectious disease specialist may treat a patient using injected antibiotics that are also used for MRSA patients. Alternatively, due to the clinical condition and the suspicion of MRSA infection in a given patient, a physician might inject antibiotics to treat MRSA before antimicrobial resistance is evaluated. Therefore, the definition of MRSA patients solely based on administration of injected anti-MRSA antibiotics (as in the HIC data) may be less specific than the definition of MRSA patients used by JANIS. This is one reason for the differences in age-specific incidence between the HIC and JANIS data. These facts support the utility of infectious disease surveillance using medical receipt data, and suggest that information from HICs is useful for evaluating the infectious disease surveillance system. Further studies should evaluate the sensitivity and specificity of the diagnostic criteria for MRSA patients based on information in HICs. Similarly, more appropriate diagnostic criteria for MRSA patients should be identified by considering the length of stay, the amounts of antibiotics given for MRSA infection, and the diagnosis. Differences in the sensitivity and specificity of various diagnostic criteria [29, 30] must be considered in order to appropriately evaluate MRSA surveillance using data from HICs.

This study has two major strengths. First, it evaluated the quality of sentinel surveillance using a data source that was not based on reports from physicians. The surveillance system substantially underestimates the number of MRSA cases because for various reasons, they are not all reported by physicians [7]. Thus, it is necessary to verify that any change in the reported incidences of specific diseases is due to the actual decline of the disease, rather than a failure of surveillance. Our finding that notification to JANIS about MRSA patients was not affected by patients' age suggests the usefulness of information from HICs for the evaluation of the infection surveillance system. Previous surveys have studied the reliability of the MRSA surveillance system by asking medical facilities to report additional information [29, 31]. However, the reliability of such investigations are affected by response rates, because data are not obtained from medical institutions that do not report to the surveillance system. Our method used data from sources other than direct physician notifications to the surveillance system. It was reported that the sentinel surveillance system for adult measles in Japan was lower than the number of measles cases identified in HICs [7]. The above facts indicate the usefulness of evaluating the surveillance system using HIC-derived information.

The second strength of this study is that the incidence of MRSA inpatients could be calculated using a standardized definition in a specific population. The conventional infectious disease surveillance system in Japan is based on the number of patients reported by sentinel medical facilities. Since patients' names and addresses may not necessarily be reported, the current surveillance system cannot distinguish patients who visit multiple medical facilities. The health insurance system in Japan allows patients to visit any medical facility without first consulting generalists, and thus the number of patients reported by medical facilities does not necessarily reflect the population surrounding any given facility. The number of patients reported by each sentinel medical facility is affected not only by the incidence of the target disease but also by the number of patients or the population where the medical facility is located. To compare epidemic patterns in different areas [32–34] or across different time spans, incidence is more appropriate than the number of MRSA cases per patient-day [23] or device-day [23, 35] in each medical facility, because it simultaneously considers the number of patients (the numerator) and the total population (denominator).

There are two major limitations to this study. The first is that HICs do not provide information on the degree of drug resistance. Because HICs are designed solely to claim the costs of all healthcare services offered by a healthcare provider in a given month, the results of laboratory tests may not be accessible even when we know that such tests have been performed. In this study, when using the HIC data, MRSA patients were defined as those injected with anti-MRSA drugs; this definition does not consider the actual drug resistance of pathogens isolated from patients. By contrast, JANIS defines MRSA infection more specifically based on results of antimicrobial susceptibility tests. MRSA bloodstream infection rates obtained using laboratory-identified event-reporting modules differ from those obtained from traditional surveillance [36]. The difference in diagnostic criteria is one reason for the discrepancy between HICs and JANIS in the number of reported MRSA cases. However, MRSA treatment guidelines strongly restrict anti-MRSA drug injections [27, 28], and the established use of laboratory drug resistance tests prior to anti-MRSA drug injections was initiated only recently.

The second limitation is that we examined only information regarding anti-MRSA medicine use and patients' age. Thus, diagnoses other than MRSA infection, differences between community-acquired and nosocomial infections, disease severity, sex, and region were not evaluated in this study. Moreover, we cannot reliably estimate the rate or proportion of patients identified as having MRSA during hospitalization in all who are hospitalized because we do not have data of the number of persons who are hospitalized. However, the definition of MRSA patients from information on HICs was not affected by the reliability of the diagnoses described on HICs or uncoded diagnoses. For JANIS surveillance, precise information such as infection site and the site where the pathogen was detected are reported. In other MRSA surveillance systems, information about the drug resistance of the detected bacterium, infection site, surgical procedure, and use of central venous catheters are collected [35, 37–39]. HICs are prepared by healthcare providers in Japan for reimbursement of their services. In Japan, not all hospitals are certified for DPC/PDPS. Information about the diagnosis that caused hospitalization is reported in HICs from certified, but not uncertified, hospitals. DPC/PDPS HIC records contain information not only about health insurance qualification status, healthcare costs, clinical procedures, but also about the diagnosis that caused hospitalization and the severity of disease. This type of HIC data allows for research such as a previous study that examined the association between hospital case volume and mortality in non-elderly pneumonia patients [12]. Further studies are necessary to assess the effects of factors not analysed in this study for the relationship between age group and MRSA incidence captured in JANIS and HIC datasets.

## CONCLUSION

This study used information from HICs and JANIS to estimate the age-specific incidence of MRSA inpatients for a 1-year period in Japan. The incidence ratios were constant between the two datasets for all age groups, suggesting that JANIS notification of MRSA cases was not affected by patients' age.
